# The Smithsonian Institution

**Published:** 1855-02

**Authors:** 


### EDITORIAL DEPARTMENT.

*The Smithsonian Institution.*—The recent session of the Board of Regents of this national chapel of ease, has terminated in a manner likely to bring about an explanation of its affairs. Although the Board has duly sanctioned and endorsed all the actions of its secretary, it has been done in such an evident spirit of favoritism that the Hon. Rufus Choate, its most distinguished and well-informed member, has thrown up his office in disgust, and in a manly and spirited letter to the two Houses of Congress, calls on them to define the law governing the institution. While the difference of opinion relative to the interpretation of the law is thrown out to the country as the real issue, it is evident to those who have any means of arriving at the truth, that there is a more personal issue, viz: whether secretary Henry is or is not to be the one man power in the control of the Institution, and whether his arbitrary will and personal ambition is to override the formal acts of Congress.

Let us examine first the resolution of the Regents sanctioning Prof. Jowett's expulsion from his office as Librarian.

Mr. Pearce offered the following:

"The secretary having stated to the Board that since the last meeting of the Regents in 1854, he had removed Mr. Jewett, under the authority declared to be vested in him by the resolution of July 8, 1854.

"Resolved, That while the Board regret the necessity of Mr. Jewett's removal, they approve of the act of the secretary.

"Resolved, That this approval by the Board is not deemed by them to be essential to the validity of the act of the secretary in so removing Mr. Jewett."

These resolutions were passed, eight in favor, to two voting nay.

The preamble declares Prof. Jewett's expulsion to have been made by secretary Henry under authority vested in him by the Regents, July 8, 1854. It is as evident, then, as cause and effect can be, that the resolution of July 8, 1854, was for the purpose of placing Prof. Jewett under the power of the secretary, inasmuch as the expulsion followed the resolution almost immediately. Now what was Prof. Jewett's crime?

Under the act of Congress he had the control of $25,000, or five-sixths of the income, for the purpose of building up a library. The Regents had no legitimate control of this expenditure; the simple letter of the law requiring the purchase of books and articles for the museum to that amount. Of course this crippled Prof. Henry's projects very much, and he accordingly set to work to break up the library system. *But not by amending the law —that was too doubtful an expedient.*

Prof. Jewett was disposed to be conciliatory. Under the earnest solicitations of those professors connected with the department of physics, viz: Baird, Guyot and Blodget, he conceded much to the pressing demands of research. He accepted $15,000 for library and museum both, when the law gave him $25,000 for the library alone. This was generous, but he had conceded too much; Prof. Henry cut him down to $12,500 the next year, and during the last year the wedge was driven up to its base, and no appropriation at all was made to the library. Stripped thus of all usefulness in his post, Prof. Jewett made an indignant defence, but his ruin was complete; and under the authority given to Prof. Henry by the Regents *for that express purpose*, he was summarily dismissed.

In order to give some color of justice to this arbitrary proceeding, it was necessary, before fully developing his intentions, for Prof. Henry to devise some apparently useful plan of expenditure for the money thus plundered from the library. Accordingly, in 1851, Prof. Lorin Blodget was called to take charge of a meteorological department. The original design was to establish, at different points, a small number of expensive observatories, where sundry scientific gentlemen should have a comfortable berth with good pay·

To this Prof. Blodget objected, and finally carried his point. Relying entirely on the enthusiasm of amateurs, he soon built up a system of observations the most extensive, reliable and valuable ever attained in any country. Indeed, the intrinsic worth of this system became so evident to the scientific world, that Prof. Blodget was rapidly becoming the star of the Institution. He was growing too important, and sundry little checks were put in his way to prevent his fame from belittling that of the secretary.

The final disposition of the impracticable Prof. Blodget is summed up by the Regents in this wise:

"Mr. Pearce, in behalf of the Executive Committee, presented the following Report in relation to the case of Mr. Blodget, which had been referred to that committee by the Board:

"*Report*—At a meeting of the Board of Regents, held Saturday, July 8, 1854, the Executive Committee was authorized to investigate and settle the business presented to the Board by the secretary, in reference to the adjustment of the claims of Mr. Lorin Blodget. The committee having investigated the matter referred to them, present the following report, in part: Mr. Blodget was employed by the secretary of the Institution to aid him, by such labors, in relation to the meteorological observations, under the direction of the Smithsonian Institution, as the secretary might assign. The rates of compensation for these services were fixed from time to time by the same officer, and Mr. Blodget is entitled to no other compensation than that paid to him. His footing in the institution was simply that of a temporary employee of the secretary, in whose hands rested the determination of his duties, pay and duration of service. Employed and paid for these services, in connection with the meteorological operations, the fruits of his labors belong exclusively to the Institution. In addition to these payments, the committee is prepared, on receiving satisfactory statements or vouchers from Mr. Blodget, of reasonable expenses incurred during any journeys he may have made, with the consent of the secretary, for objects connected with his duties in meteorology in the Institution, to refund the amount; as also, any moneys which may appear to the satisfaction of the committee to have been paid out by him, and not already repaid, for clerical or other services connected with the meteorological observations of the Smithsonian Institution, and for which an equivalent advantage has been received.

J. A. PEARCE,
J. G. TOTTEN,
A. D. BACHE,
Executive Committee.

"Communications, and a memorial from Mr. Blodget to the Board, was then read, and ordered to lie on the table.

"The report of the Executive Committee was then adopted unanimously."

So Mr. Blodget was to *aid* the secretary! Scientific men who have had business in the meteorological department, will learn here, for the first time, that Prof. Henry had anything whatever to do with it. We state it as a matter within our personal knowledge, that *all* the scientific correspondence in that department has been maintained by Prof. Blodget, and that he, and he only, has ever attempted to classify or investigate the splendid mass of statistics collected by the department. The little which has been published has been through him, and why it has not been more may be very readily explained.

The whole secret of Prof. Blodget's failure is briefly this: He found that the opposition made by Prof. Henry to the library covered some personal ends, to which he declined to lend himself. Again, he was building up a reputation for himself.

Now, if Prof. Blodget was employed simply "in a clerical capacity, as an aid to the secretary," of course it was an easy matter to get rid of him. It only remained to pay him off and discharge him. But this was not the plan adopted by the omnipotent secretary. We consider as pretty good evidence that Prof. Blodget was not legally under the power of the secretary, the fact that Prof. Blodget, on returning from dinner to his room in the Smithsonian building, on the 11th of October last, found the doors barricaded, and the windows planked up by the order of the secretary, during his brief absence. No matter what was Blodget's real status in the Institution, the act was one of the most cowardly and arbitrary measures ever known in the scientific or any other decent world.

The report of the Executive Committee indicates that there has been some question as to the proprietorship of the results of Prof. Blodget's researches. So there has, and it is not to be thus cavalierly set aside. The committee indicate that inasmuch as Prof. Blodget may have incurred some little expenses in traveling, &c., it would be fair to make those right. If this means anything, it means that Blodget has a just and equitable claim which remains unadjusted. Now what is the nature of this debt? Simply, that in the payment of the current expenses of his department, Prof. Blodget has paid out some $2,500 over and above his salary. If he is only a clerk he is a most liberal one!

Prof. Blodget claims, that as these investigations have been made at his expense, and conducted by his labors, that the Institution should either pay him and print the results, or give him the results to publish himself. Thus far it has refused to do either. In the meantime the meteorological department has expired. Having fulfilled its original purpose of killing off the Librarian, it gives up the ghost and makes no sign. The laborious investigations of more than two hundred amateur observers for three years of toil, are entombed in a common mausoleum with Prof. Blodget's private books and papers, which were locked up by order of the secretary. They are lost to science, and the patient observers who have sent on their tri-daily record, might as well have burnt them up at home in their own coal grates. No man but Blodget is now capable of analyzing them, and the two hundred amateurs feel that they have been cheated and betrayed by a venal and selfish mismanagement. Of course they have ceased to forward their returns.

Behold, then, the results of this magnificent Institution! The additions to human knowledge conferred by it during the past two years, have been confined entirely to knowledge of human nature, displayed in a most uninviting form.

We have a proposition to make, which we think the best possible plan to get rid of this quarrel. Make a donation of the Smithsonian building to some colored church in Washington, and send the fund back to England to revert to the Crown. So long as it remains here in its present shape, it will be a nest of nepotism; like all other government schools confided to a Board of Regents made up of politicians, absorbed in their own purposes, or too old and careless to give any attention to its management.

